# Pilot Study: Personalized Medicine in Endoscopy, Can Pharmacogenomics Predict Response to Conscious Sedation?

**DOI:** 10.3390/jpm13071107

**Published:** 2023-07-07

**Authors:** Himesh B. Zaver, Hassan Ghoz, Balkishan Malviya, Aman Bali, Samuel Antwi, Ann M. Moyer, Yan Bi

**Affiliations:** 1 Department of Internal Medicine, Mayo Clinic, Jacksonville, FL 32224, USA; 2 Division of Gastroenterology and Hepatology, Mayo Clinic, Jacksonville, FL 32224, USAbi.yan@mayo.edu (Y.B.); 3 Department of Quantitative Health Sciences, Mayo Clinic, Jacksonville, FL 32224, USA; antwi.samuel@mayo.edu; 4 Department of Laboratory Medicine and Pathology, Mayo Clinic, Rochester, MN 55905, USA; moyer.ann@mayo.edu

**Keywords:** sedation, endoscopy, pharmacogenomics, individualized medicine

## Abstract

Background: Adequate response to moderate (conscious) sedation varies significantly between individuals. Polymorphisms in genes encoding drug metabolizing enzymes can lead to inter-individual variability in drug efficacy, potentially influencing sedation requirements during endoscopic procedures. Objectives: The aim of this study was to assess the potential role of inter-individual variation in inherited polymorphisms of drug-metabolizing enzymes, cytochrome P450 (CYP450), specifically *CYP3A4* and *CYP3A5*, in sedation requirements for outpatient endoscopic procedures. Methods: A retrospective analysis of sedation requirements and pharmacogenomics data in 106 unique patients who received outpatient esophagogastroduodenoscopy (EGD), colonoscopy, or both between December 2011 and February 2019 was conducted. Patients were divided into two groups based on their sedation requirements during endoscopy (high vs. normal sedation). Results: Patients with reduced a CYP2C19 metabolism (poor + intermediate metabolizers) (odds ratio [OR] = 0.38, 95% confidence interval [CI]: 0.16–0.91, *p* = 0.03), poor CYP3A5 metabolism (OR = 0.25, 95% CI: 0.095–0.65, *p* = 0.0046), and poor UGT1A1 (OR = 2.76, 95% CI: 1.07–7.13, *p* = 0.08) had higher odds of requiring normal sedation compared to those with CYP2C19 increased metabolism, CYP3A5 intermediate metabolism, and UGT1A1 intermediate metabolism. Conclusion: Information about inter-individual variation in (CYP450) genes may be useful for determining the sedation requirements for outpatient endoscopic procedures. We found that patients with reduced CYP2C19 metabolism, poor CYP3A5 metabolism, and poor UGT1A1 metabolism were more likely to require normal sedation requirements during outpatient endoscopic procedures.

## 1. Introduction

Patient comfort and safety are important outcome measures in the assessment of endoscopic quality and influences both patient and physician satisfaction. Outpatient endoscopic procedures are primarily completed using moderate (conscious) sedation. Cytochrome P450 (CYP450) enzymes are critical in the metabolism of many drugs, including those used during endoscopic procedures. Variations in the genes encoding these CYP enzymes can result in differences in their activity and expression, affecting metabolism and drug clearance. Preemptive pharmacogenomics testing may provide guidance on medication selection and reduce the risk of adverse reactions [1]. Fentanyl, midazolam, and meperidine are the most frequently used medications during moderate sedation and are primarily metabolized by the CYP3A4 and CYP3A5 enzymes, respectively. The dosage required to achieve optimal sedation varies between patients. Failed moderate sedation is estimated to occur in approximately 4–35% of patients [2]. Prior studies exploring pharmacogenomic influence on sedation requirements is limited. The reasons for failed moderate sedation are not completely clear, but genetic predisposition may play a role. Inter-individual variability in genes encoding drug metabolizing enzymes can lead to differences in drug efficacy, as seen with the genotype-guided dosing of warfarin, oral P2Y12 inhibitors, and proton pump inhibitors [3,4,5]. It remains to be seen if the inherited single nucleotide polymorphisms (SNPs) involved in drug metabolism could be used to guide sedation selection and dosing for outpatient endoscopic procedures. The aim of this study was to assess the potential role of inter-individual variation in the inherited polymorphisms of drug-metabolizing enzymes (CYP450), specifically *CYP3A4* and *CYP3A5*, in sedation requirements for outpatient endoscopic procedures.

## 2. Materials and Methods

We conducted a retrospective analysis of sedation requirements and pharmacogenomics data in 106 unique patients who received outpatient upper endoscopy (EGD), colonoscopy, or both, between December 2011 and February 2019 at the Mayo Clinic, Rochester, MN. Patients identified for our study were enrolled in the RIGHT 10K Study at Mayo Clinic. The RIGHT 10K Study was a genotyping study in which 84 genes from 10,000 patients were genetically sequenced to identify phenotypic variants. Our study population were those enrolled in the RIGHT 10K study who had either an EGD, colonoscopy, or both [6,7]. Patients were divided into two groups based on sedation requirements during endoscopy (high vs. normal sedation). The high sedation requirement group was defined by a sedation requirement of midazolam >10 mg, fentanyl >100 mcg, meperidine >100 mg, or an aborted procedure due to failed moderate sedation and/or the transition to propofol sedation to complete the procedure. The normal sedation requirement group was defined as the complete absence of the above conditions that define the high sedation requirement group. The normal sedation requirement is similar to practical sedation regimens from the prior literature [8,9]. Pharmacogenomic data were obtained from DNA sequencing as part of the RIGHT study [6,7]. Details of the methods used for the genotyping of germline DNA and quality control checks have been published previously [6,7]. We selected seven candidate genes involved in the drug metabolism of commonly used drugs in moderate sedation and other common medications used in combination with sedation drugs that were available in the RIGHT study for analysis: *CYP1A2*, *CYP2C19*, *CYP2C9*, *CYP2D6*, *CYP3A4*, *CYP3A5*, and *UGT1A1*. Gene phenotypes were grouped for analysis as follows: “CYP1A2 reduced metabolism” (CYP1A2 intermediate + CYP1A2 normal metabolizers) vs. “CYP1A2 increased metabolism” (CYP1A2 rapid metabolizers); “CYP2C19 reduced metabolism” (CYP2C19 poor + CYP2C19 intermediate metabolizers) vs. CYP2C19 normal metabolism vs. “CYP2C19 Increased metabolism” (CYP2C19 rapid + CYP2C19 ultrarapid metabolizers); “CYP3A4 reduced metabolism” (Poor + intermediate metabolizers) vs. CYP3A4 normal metabolizers; “CYP2D6 reduced metabolism” (CYP2D6 poor + CYP2D6 intermediate metabolizers) vs. CYP2D6 normal metabolizers vs. “CYP2D6 increased metabolism” equals CYP2D6 (ultrarapid metabolizers). The standard phenotype groups (poor metabolizer, intermediate metabolizer, and normal metabolizer) were used for *CYP2C9, CYP3A5,* and *UGT1A1* [10,11].

### Statistical Analysis and Genomic Information

Analyses were performed among 106 unique patients who underwent sedation for colonoscopy or upper gastrointestinal endoscopy. The patients were categorized as requiring normal sedation or high sedation. The distribution of patient characteristics were compared between patients requiring normal sedation and those requiring high sedation, using Student’s *t*-test for continuous variables and Fisher’s exact test for categorical variable. Genomic DNA was sequenced using the *PGRNseq version 3* capture reagent at the Baylor College of Medicine’s Human Genome Sequencing Center Clinical Laboratory. After the transfer of data, the Mayo Clinic Personalized Genomics Laboratory interpreted the genotype and phenotype data using standard clinical laboratory practices. CYP2D6 genotypes were determined using the specialized software CNVAR v.1.0, which was developed and clinically validated at the Mayo Clinic. For each gene, rare variants were classified by pharmacogenomics experts following a modified version of the American College of Medical Genetics and Genomics and the Association for Molecular Pathology (ACMGG/AMP) Guidelines for variant interpretation, as described in detail elsewhere [12]. The normal metabolizer phenotype for each respective gene was chosen as *“reference”*. It should be noted that the normal metabolizer phenotype is not necessarily the most common phenotype associated with each gene. For association analyses, unconditional logistic regression was used to calculate the odds ratios (ORs), the associated 95% confidence intervals (CIs), and the *p*-values for the relative odds of requiring high sedation. Analyses were performed in multivariable-adjusted models, adjusting for age (continuous), gender, race (White, Black, other), and body mass index (BMI) (continuous). All statistical tests were two-sided. All analyses were performed with SAS version 9.4, with *p*-values < 0.05 being considered statistically significant. Descriptive statistics for continuous variables, such as subject characteristics and basic demographics, were reported as means, median, and standard deviations. Categorical variables were summarized with the number and percentage of patients. Comparisons between the high sedation (HSD) group and normal sedation (NSD) group were performed using the Wilcoxon rank sum test or Fisher’s exact test. Continuous phenotypes assuming normality were measured using Student’s *t*-test. Institutional Review Board (IRB) approval was obtained. Patients were not compensated in any form for participating in this study.

## 3. Results

A total of 106 patients were included in the study, among whom 212 procedures were completed. Of these procedures, 89 required HSD and 123 required normal sedation NSD requirements. The NSD group were slightly older than the HSD group (73 years vs. 67 years, *p* ≤ 0.0001); however, the two groups did not differ by sex, race, BMI, number of polyps removed, or number of biopsies. The total duration of procedures was longer for procedures requiring high sedation compared to normal sedation (median 18 min vs. 24 min, *p* = 0.012) (Table 1). After adjusting for age, sex, race, and BMI, multivariate analysis supported that three gene phenotypes were found to be significantly associated with the normal sedation requirement (Table 2). Patients with reduced CYP2C19 metabolism (poor + intermediate metabolizers) (OR = 0.38, 95% CI: 0.16–0.91, *p* = 0.03), poor CYP3A5 metabolism (OR = 0.25, 95% CI: 0.095–0.65, *p* = 0.0046), and poor UGT1A1 (OR = 2.76, 95% CI: 1.07–7.13, *p* = 0.08) had higher odds of requiring normal sedation compared to those with CYP2C19 increased metabolism, CYP3A5 intermediate metabolism, and UGT1A1 intermediate metabolism (Table 2).

## 4. Discussion

Our pilot study suggests that inter-individual variation in cytochrome P450 (CYP450) genes may be useful for determining sedation requirements for outpatient endoscopic procedures. We found that patients with reduced CYP2C19 metabolism, poor CYP3A5 metabolism, and poor UGT1A1 metabolism were more likely to require normal sedation requirements during outpatient endoscopic procedures. These findings illustrate the potential utility of the preemptive testing of relevant genes to determine the inter-individual variation of moderate sedation requirement.

To date, there are more than fifty *CYP450* enzymes that have been discovered. *CYP1A2*, *CYP2C9*, *CYP2D6*, *CYP3A4*, and *CYP3A5* are responsible for up to 90% of drug metabolism [11]. CYP3A4 and CYP3A5, specifically, are responsible for the metabolism of 45–60% of prescribed drugs, including medications used for moderate sedation, such as midazolam, fentanyl, and meperidine [13]. Thirty-five *CYP3A4* alleles and nine *CYP3A5* alleles have been identified so far [10,14]. Different phenotypes based on allele function (normal-, intermediate-, poor-, rapid-, ultrarapid- metabolizer) may affect serum concentration leading to response variability.

Both midazolam and fentanyl’s therapeutic activity is primarily due to parent drug activity and not drug metabolites. In practice, a patient identified with reduced CYP3A4 metabolism (poor + intermediate metabolizers), and/or CYP3A5 poor metabolizers, theoretically may have higher serum concentrations of the active drug due to the poor metabolism of the parent drug. It may be considered to use lower concentrations of medication to reach therapeutic levels to avoid potential adverse reactions to moderate sedation. Our data supports further investigation of this notion. Other commonly prescribed medications metabolized by CYP3A4 and CYP3A5 include antimicrobials, such as macrolide antibiotics, HIV antivirals, statins, corticosteroids, and calcium channel blockers [13,14,15].

UGT1A1 is an enzyme and member of the UDP-glucuronosyltransferase (UGT) enzyme family, which are responsible for the glucuronidation of various substances and drugs in the body (primarily anticancer drugs) [16]. CYP2C19 is an enzyme responsible for the processing of the 10% most prescribed drugs (proton pump inhibitors, clopidogrel, antiepileptic drugs, antidepressants) but not directly related with fentanyl or midazolam metabolism [17,18]. Patients with reduced CYP2C19 metabolism (poor + intermediate metabolizers) and reduced UGT1AI showed higher odds of requiring NSD. This may be due to UGT1A1 substrate inhibition or complex drug metabolism pathways where drug function is regulated by multiple CYP, where uptake, distribution, and excretion are frequently modified by other medications, herbs, food intake, and native hepatic and renal function. Pharmacogenomic information as a clinical tool may assist in identifying patients at risk for medication failure. Such information may be used by providers to guide future medication use and educate patients on multifactorial interactions, including medication–gene interactions that can affect medication efficacy.

We recognize that our pilot study has limitations. Our study was conducted at a single quaternary referral center and was limited by sample size and race demographics; therefore, larger studies are needed to verify these findings. Future studies at other centers evaluating changes in sedation requirements with respect to the training level of endoscopist and native hepatic and renal function could add valuable insight. Mechanistic studies of the polymorphisms of genes, including the impact of environmental factors, are needed. The application of preemptive pharmacogenomic testing does have its challenges. The combinations of different gene phenotypes within individuals may make it difficult to interpret genotyping results if the results for some phenotypes suggest higher odds of normal sedation and others of higher sedation or vice versa. There are limitations of private insurance and government insurance programs to reimburse testing. As a clinical tool, pharmacogenomic testing for the most part has been limited to academic medical centers. This is due, in part, to provider knowledge of the topic, the ability to interpret data, and the hesitancy of interrupting provider workflow. These preliminary findings, however, describe the potential benefit of applying individual genetic information to help determine the sedation dosage in patients undergoing outpatient endoscopic procedures. It should also be noted that these CYP enzymes also metabolize other commonly prescribed medications, and dose adjustments for those medications may be considered. Providing a more personalized and safer drug therapy may improve patient satisfaction and comfort during outpatient endoscopic procedures. Preemptive pharmacogenomic testing may help clinicians make a more informed decision when selecting the most appropriate drugs and dosing regimens for their patients based on the individual’s genetic background.

## Figures and Tables

**Table 1:** Distribution of patient characteristics and respective sedation requirements.

	Normal Sedation (N = 123)	High Sedation (N = 89)	Total (N = 212)	*p* Value
**Age at time of procedure**				<0.0001
Mean (SD)	73.1 (7.3)	67.8 (8.8)	70.9 (8.4)	
Median	73.9	69.6	72.5	
Q1, Q3	70.3, 76.5	62.7, 74.8	67.8, 76.2	
Range	(43.6–116.5)	(46.4–80.1)	(43.6–116.5)	
**Patient sex**				0.5149
F	58 (47.2%)	46 (51.7%)	104 (49.1%)	
M	65 (52.8%)	43 (48.3%)	108 (50.9%)	
**Race**				0.8174
White	122 (99.2%)	88 (98.9%)	210 (99.1%)	
Mixed	1 (0.8%)	1 (1.1%)	2 (0.9%)	
**BMI**				0.5614
N	123	89	212	
Mean (SD)	28.1 (6.1)	27.6 (9.7)	27.9 (7.8)	
Median	28.0	28.3	28.0	
Q1, Q3	25.1, 31.3	26.2, 31.2	25.5, 31.2	
Range	(0.0–52.5)	(0.0–66.6)	(0.0–66.6)	
**Procedure**				0.1546
Colonoscopy	86 (69.9%)	70 (78.7%)	156 (73.6%)	
EGD	37 (30.1%)	19 (21.3%)	56 (26.4%)	
**Which procedure came first**				0.3127
Colonoscopy	100 (81.3%)	77 (86.5%)	177 (83.5%)	
EGD	23 (18.7%)	12 (13.5%)	35 (16.5%)	
**Polyp**				0.4020
Missing	33 (26.8%)	17 (19.1%)	50 (23.6%)	
No	22 (17.9%)	16 (18.0%)	38 (17.9%)	
Yes	68 (55.3%)	56 (62.9%)	124 (58.5%)	
**Number of polyps**				0.1618
N	68	56	124	
Mean (SD)	1.9 (1.1)	2.2 (1.3)	2.0 (1.2)	
Median	2.0	2.0	2.0	
Q1, Q3	1.0, 2.5	1.0, 3.0	1.0, 3.0	
Range	(1.0–5.0)	(1.0–7.0)	(1.0–7.0)	
**Biopsies**				0.0817
Missing	68 (55.3%)	60 (67.4%)	128 (60.4%)	
No	10 (8.1%)	2 (2.2%)	12 (5.7%)	
Yes	45 (36.6%)	27 (30.3%)	72 (34.0%)	
**Total duration of procedures (minutes)**				0.0102
N	118	89	207	
Mean (SD)	20.8 (13.8)	24.6 (12.2)	22.4 (13.3)	
Median	18.0	24.0	21.0	
Q1, Q3	9.0, 29.0	16.0, 31.0	12.0, 30.0	
Range	(3.0–68.0)	(4.0–70.0)	(3.0–70.0)	

**Table 2:** Multivariable-adjusted association between drug metabolism gene phenotypes and sedation requirement during endoscopic procedures.

	Normal (N = 123)	High (N = 89)	OR (95% CIs)	*p* Value ^a^
***CYP1A2***				
Reduced metabolism (intermediate + normal metabolizers)	8	9	1.00 (ref)	
Increased metabolism (rapid metabolizers)	115	80	0.16 (1.16–1.34)	0.16
***CYP2C19***				
Normal metabolizer	53	40	1.00 (ref)	
Reduced metabolism (poor + intermediate metabolizers)	32	11	**0.38 (0.16–0.91)**	**0.03**
Increased metabolism (rapid + ultrarapid metabolizers)	38	38	1.16 (0.60–2.22)	0.66
***CYP2C9***				
Normal metabolizer	77	52	1.00 (ref)	
Intermediate metabolizer	46	37	1.20 (0.66–2.19)	0.55
***CYP2D6***				
Normal metabolizer	39	32	1.00 (ref)	
Reduced metabolism (poor + intermediate metabolizers)	81	57	0.80 (0.43–1.47)	0.47
Increased metabolism (ultrarapid metabolizers)	3	0	NE	
***CYP3A4***				
Normal metabolizer	112	79	1.00 (ref)	
Reduced metabolism (poor + intermediate metabolizers)	11	10	1.57 (0.61–4.03)	0.34
***CYP3A5***				
Intermediate metabolizer	8	16	1.00 (ref)	
Poor metabolizer	115	73	**0.25 (0.095–0.65)**	**0.0046**
***UGT1A1***				
Normal metabolizer	61	35	1.00 (ref)	
Intermediate metabolizer	51	41	1.65 (0.87–3.13)	0.12
Poor metabolizer	11	13	**2.76 (1.07–7.13)**	**0.08**

^a^ Each gene phenotype was modeled independently, adjusting for age at the time of procedure (continuous), sex, race (White, Black, other), and body mass index (continuous). Abbreviation: confidence interval (CI), reference (ref), not estimable (NE).

## Data Availability

Available upon request.

## Abbreviations

EGD	Esophagogastroduodenoscopy
CYP450	Cytochromes P450
PGRN	Pharmacogenomics Research Network
SNPs	Single nucleotide polymorphisms
OR	Odds ratio
CI	Confidence interval
BMI	Body mass index
IRB	Institutional Review Board
HSD	High sedation
NSD	Normal sedation
